# Loss of calponin h1 confers anoikis resistance and tumor progression in the development of high-grade serous carcinoma originating from the fallopian tube epithelium

**DOI:** 10.18632/oncotarget.18024

**Published:** 2017-05-19

**Authors:** Kai-Hung Wang, Sung-Chao Chu, Tang-Yuan Chu

**Affiliations:** ^1^ Department of Research, Center for Prevention of Gynecological Cancer, Buddhist Tzu Chi General Hospital, Hualien, Taiwan; ^2^ Institute of Medical Sciences, Tzu Chi University, Hualien, Taiwan; ^3^ Department of Hematology and Oncology, Buddhist Tzu Chi General Hospital, Hualien, Taiwan; ^4^ Department of Obstetrics and Gynecology, Buddhist Tzu Chi General Hospital, Hualien, Taiwan

**Keywords:** fallopian tube epithelium, high-grade serous carcinoma (HGSC), ovarian cancer, Calponin h1 (CNN1), anoikis

## Abstract

Increasing evidence indicates that ovarian high-grade serous carcinoma (HGSC) originates from the fallopian tube epithelium and metastasizes to the ovary as the secondary site. A working hypothesis is that detached tubal HGSC cells survive anoikis and implant on the ovary. In this study, we found that downregulation of calponin h1 (CNN1) is necessary for the anoikis survival and cell transformation. CNN1 was progressively downregulated in cells and tissues representing different stages of HGSC development from fallopian tube epithelium (FTE). Knock down of CNN1 in immortalized human FTE cells conferred gains of resistance to anoikis and transformation phenotypes including anchorage independent growth (AIG) and xenograft tumorigenesis in NSG mice. Conversely, overexpression of CNN1 in RAS-transformed FTE cells resulted in an almost complete loss of AIG and tumorigenesis. Besides, there was a dramatic change of cell morphology from a polygonal, raised appearance to a round and flattened one. Increase in cell adhesion to laminin and collagen, and reduction in cell motility, anoikis resistance and invasiveness were also observed. A microarray analysis revealed upregulation of genes involved in cytoskeleton stabilization and signal transduction, and downregulation of genes involved in cytokine and chemokine activities. The study disclosed multiple tumor suppressor roles of CNN1 in the development of HGSC from the fallopian tube, and loss of CNN1 expression is crucial for its metastasis to a new site.

## INTRODUCTION

Every year, approximately 225,500 women are diagnosed with ovarian cancer, with 140,200 resulting deaths [1]. Among the ovarian cancer subtypes, high-grade serous carcinoma (HGSC) is the most prevalent and lethal, representing more than 70% of ovarian carcinomas and claiming 114,000 lives worldwide each year. The high mortality of HGSC is mainly due to its elusive origin and aggressive natural history [2]. Most ovarian HGSC cases present with established carcinomatosis involving the peritoneum of almost all of the abdominal and pelvic organs. Recent detailed examinations of prophylactically removed fallopian tubes and ovaries of *BRCA1* and *BRCA2* mutation carriers have shown frequent serous tubal intraepithelial carcinoma (STIC) and early invasive carcinomas in the fimbriae of the tube, but these early lesions have never been found in the ovary [3–7].

Because they originate in the fallopian tube fimbriae, STIC cells must detach from the primary site and metastasize to peritoneal and ovarian surfaces to establish the typical advanced HGSC lesions. To achieve this, intraepithelial carcinoma cells must change from a polarized, adhesive phenotype to a pleomorphic, nonadhesive and migratory one. Indeed, STIC cells are frequently found exfoliating from the fimbrial epithelium in cell clusters, and for the most part, these clusters are not associated with cell degeneration [8], suggesting the acquisition of resistance to detachment-associated cell death or anoikis. The mechanisms underlying these phenotypic changes are unknown.

Considering that actin cytoskeletal disorganization is vital in cell metastasis [9], we assume that calponin h1 (CNN1), one of the family of actin-binding proteins that stabilize the filaments of actin and modulate various cellular biological phenotypes [10], may play a major role in the detachment of STIC cells. CNN1 is thought to play an essential role in stabilizing actin stress fibers because it can (1) bind to the thin filament of actin, tropomyosin, and calmodulin [11, 12]; (2) inhibit the actin-activated myosin ATPase [13]; (3) inhibit Ca2^+^-dependent mobility of actin on immobilized myosin [14]; and (4) induce conformational changes in actin filament [15]. CNN1 also plays a vital role in the maturation of blood vessels, metastasis, and peritoneum dissemination of different cancer cells [16–19]. In addition, CNN1 was downregulated in uterine leiomyosarcoma and may play a role as a tumor suppressor [20, 21].

To clarify the role of CNN1 in the development of HGSC arising from the fallopian tube, we characterized the expression and functions of CNN1 in the transformation of fimbrial epithelium to HGSC. We discovered a tumor suppressor role of CNN1 that must be downregulated to encourage cell exfoliation, migration, anchorage-independent growth (AIG), and tumorigenesis.

## RESULTS

### Transformation of human FTE cells with RAS^V12^

**Figure 1:**
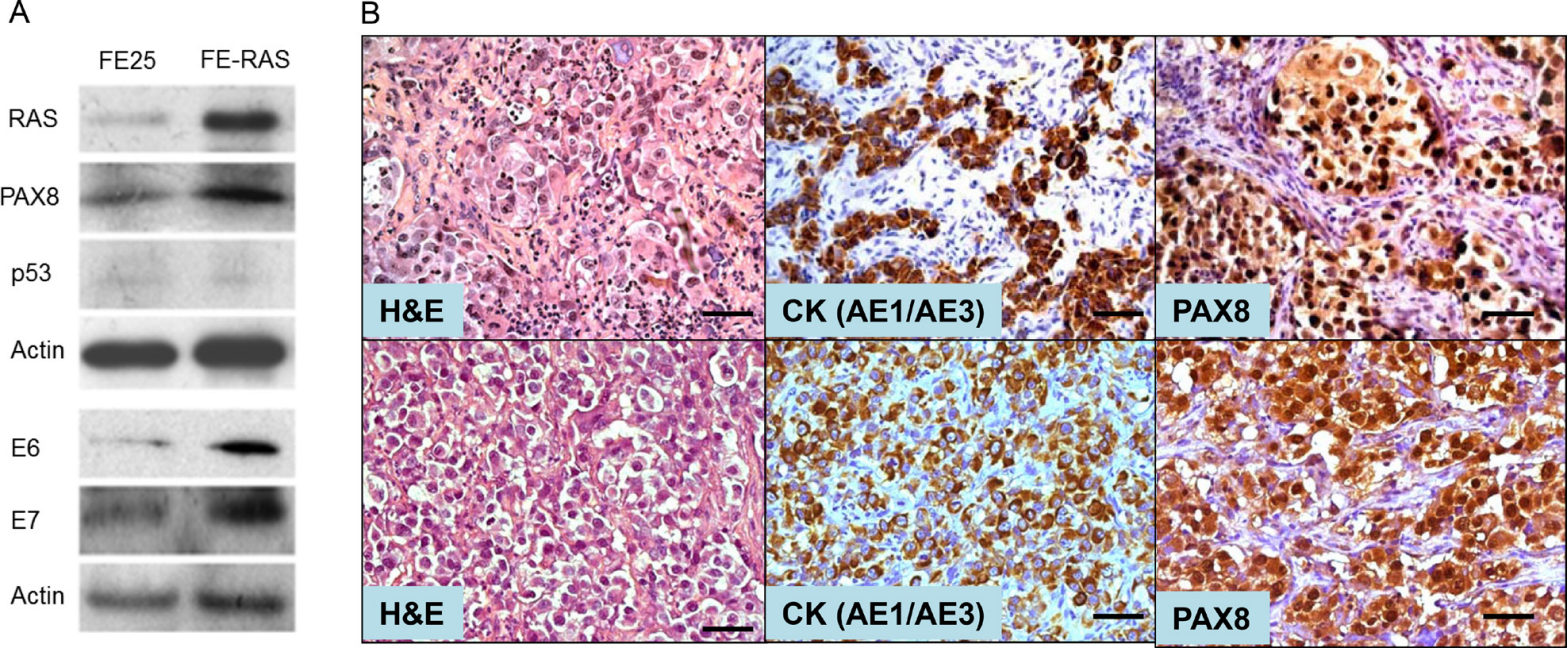
Characterization of a RAS-transformed human fimbria epithelial cell line (**A**) Human fimbria epithelial cells (FTE) were primarily cultured, transduced with pLenti-E6/E7 and pLenti-hTERT. The immortalized cell line FE25 was derived. By additional transduction with pLenti-Ras^V12^ oncogene, FE-RAS cells were derived. Western blot results showed the expression pattern of RAS, PAX8, p53, HPV E6 and E7 in FE25 and FE-RAS cells. (**B**) Immunohistochemistry of xenotumors from FE-RAS cells subcutaneous (upper panel) and intraperitoneal (lower panel) injections into NSG mice. Scale bars: 50 μm.

The PI3K/RAS pathway is one of the major oncogenic signals of ovarian HGSC, and it has been found to be altered in 45% of ovarian HGSC [22]. To investigate the role of CNN1 in the development of HGSC originating from the fallopian tube, we transformed a previous established HPV16 E6/E7-immortalized human fallopian tube FTE cell line (FE25) [23] with oncogenic RAS^V12^ and named it FE-RAS cells. As expected, FE-RAS, like FE25, expressed E6, E7 and the fallopian tube secretory cell marker PAX8, and p53 was silenced in both cells by E6 (Figure 1A). In contrast to FE25 cells, the FE-RAS cells exhibited AIG activity and grew tumors in immune-compromised mice in both subcutaneous (Figure 1B, upper panel) and intraperitoneal (Figure 1B, bottom panel) injections. The tumors had a histology of poorly differentiated adenocarcinoma, expressing human epithelial pancytokeratin (CK) and PAX8 (Figure 1B).

### CNN1 was downregulated in the development of HGSC from the fallopian tube epithelium

**Figure 2:**
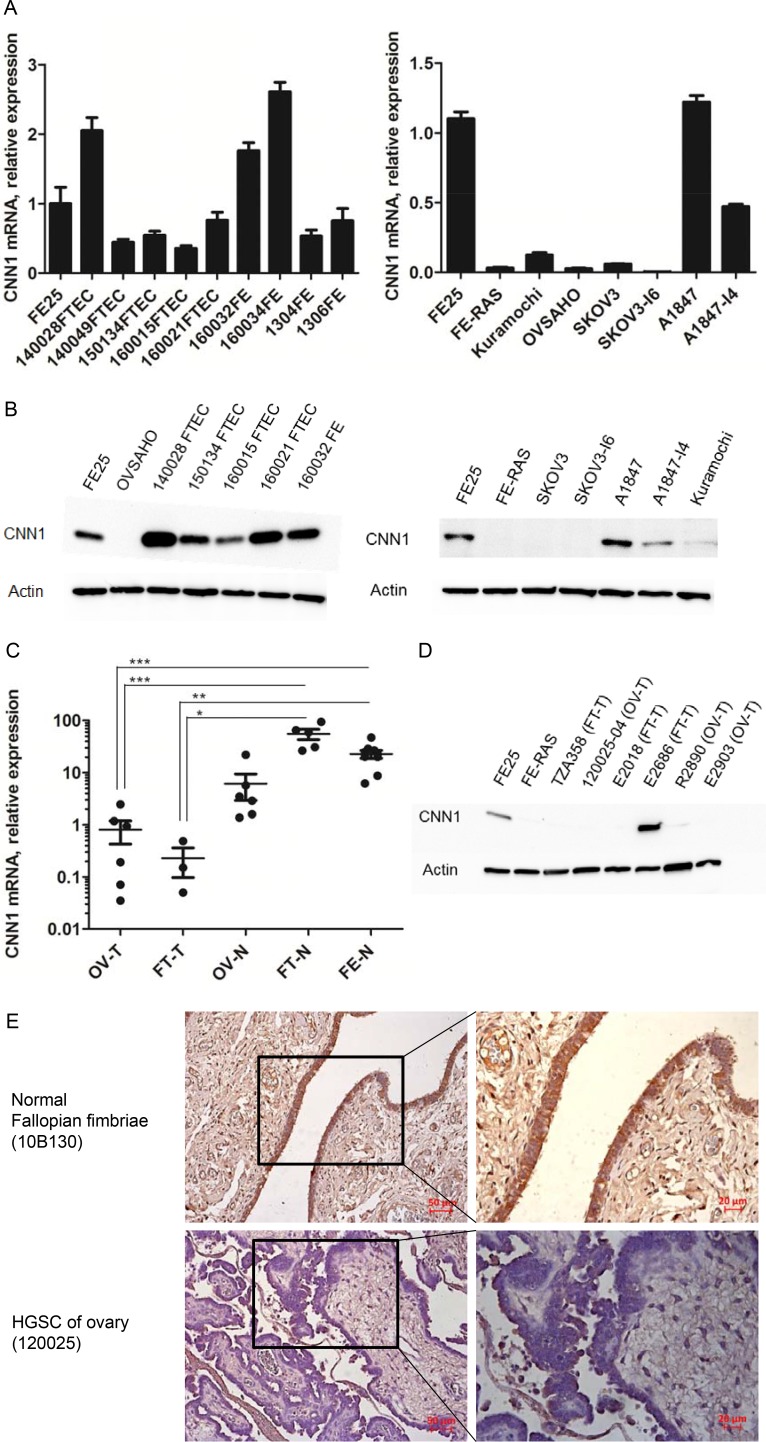
CNN1 is downregulated in the development of HGSC and in invasion progression of ovarian cancer cells (**A**) Quantitative RT-PCR and Western blot (**B**) analysis of CNN1 in primarily cultured fimbriae epithelial cells (FTEC, with the case number given), fimbria epithelial scrapings (FE, with case number given as prefix), as well as in immortalized (FE25) and transformed (FE-RAS) fimbria epithelial cell lines, two ovarian HGSC cell lines (Kuramochi, OVSAHO), and two common ovarian cancer cell lines (SKOV3, A1847) and their invasive subclone (I6 and I4). (**C**) Quantitative RT-PCR analysis of CNN1 in tissues of ovarian HGSC (OV-T, *n* = 6), fallopian tube HGSC (FT-T, *n* = 3), normal ovary (OV-N, *n* = 6), normal fallopian tube fimbria (FT-N, *n* = 5) as well as scrapings of normal fimbria epithelium (FE-N, *n* = 9). *P-value < 0.05, **P-value < 0.01, ***P-value < 0.001. (**D**) Western blot analysis of CNN1 expression in clinical HGSC specimens derived from ovary (OV-T) and fallopian tube (FT-T). (**E**) Immunohistochemistry analysis of CNN1 expression in normal fallopian fimbriae and HGSC tissues.

As shown in Figure 2A and 2B, CNN1 was highly expressed in the FE25 cells as well as in the five primary FTE cells and four fimbriae epithelial scraping cells, but was poorly expressed in the transformed FE-RAS cells, in two HGSC cell lines, namely OVSAHO and Kuramochi. In a traditional epithelial carcinoma cell line A1847, CNN1 was less expressed in the more invasive subline A1847-I4. This was also true in the mRNA level of a non-HGSC type ovarian cancer cell SKOV3 and its invasive subline SKOV3-I6. The downregulation of CNN1 in malignant transformation was also observed in clinical specimens in which HGSC tumor tissues from both the ovary and the fallopian tube had a lower expression level than did those from the normal ovary, normal fallopian tube, and fallopian tube epithelial scrapings (Figure 2C and 2D). In immunohistochemistry study, CNN1 protein is highly expressed in the epithelium of normal fallopian tube fimbria but not expressed in the ovarian HGSC tissues (Figure 2E).

### Knock down of CNN1 in immortalized FTE cells results in anoikis resistance, AIG, and xenograft tumorigenesis

**Figure 3:**
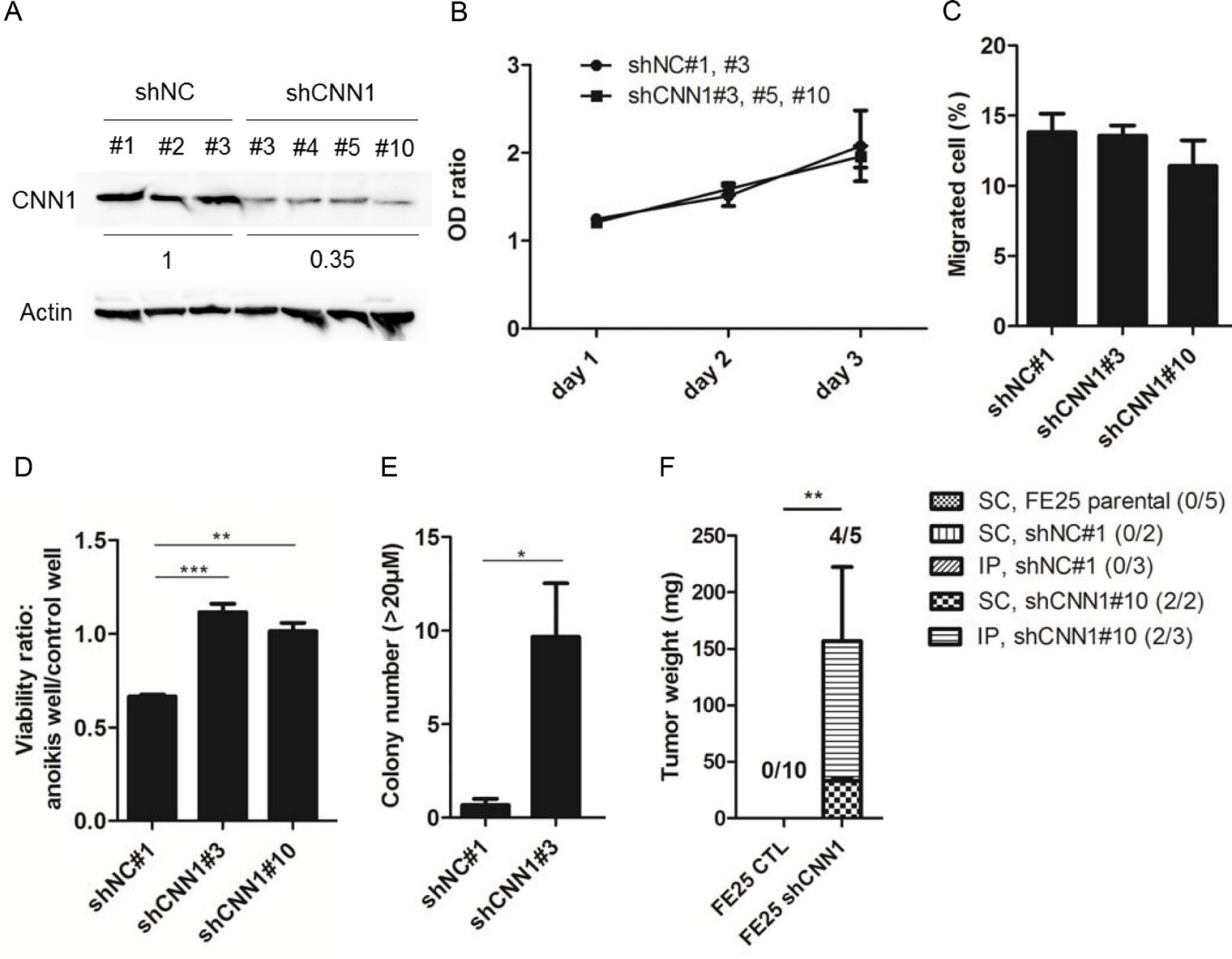
Knock down of CNN1 in FE25 cells enhanced anoikis-resistance, colony formation and xenograft tumor formation but not the cell motility Stable clones of FE25 cells transfected with shRNA of CNN1 (shCNN1) or negative control (shNC) were analyzed for protein expression (**A**), cell proliferation (**B**), Transwell migration (**C**), cell anoikis (**D**), anchorage-independent growth of colony with diameter > 20 μm (**E**) and xenograft tumorigenesis in NSG mice (**F**) as stated in Materials and Methods. In the anoikis assay, two independent stable clones of shCNN1 (shCNN1#3 and shCNN1#10) were analyzed. In AIG assay, colonies with diameter larger than 20 μm were counted. Xenograft tumors were observed 5.5 month after s.c. or i.p. injection of CNN1 knock-downed FE25 cells (FE25 shCNN1) (*n* = 5, 2 with s.c, 3 with i.p.) or CNN1-normal FE25 cells (FE25 CTL) (*n* = 10, 5 transfected with shNC, 5 without). Three of the shNC-transfected FE25 cells were i.p. injected. The others were s.c. injected. *p < 0.05, **p < 0.01, ***p < 0.001.

To explore the role of CNN1 in transformation of fallopian tube fimbrial epithelium, FE25 cells were transfected with the control shRNA or CNN1 shRNA where CNN1 protein expression was reduced to an average of 35% in different clones (Figure 3A). As shown in Figure 3B and 3C, neither cell proliferation nor cell motility was affected by CNN1 knockdown. However, the cells demonstrated superior survival in the low-attachment culture (Figure 3D), exhibited a more effective AIG (Figure 3E), and were able to grow tumors in xenograft. Compared with forming the large (> 50 μm) AIG colonies of the fully transformed FE-RAS cells, those of the CNN1-knockdown FE25 cells were relatively small (20–25 μm). The xenograft tumors grew slowly, taking 5.5 months to grow to a measurable size in both the s.c. (2 tumors in 2 injected mice) and i.p. (2 in 3 injected mice) injections in NSG mice. By contrast, cells transfected with control shNC and parental FE25 did not grow any tumors (*n* = 10, Figure 3F). Thus, knock down of CNN1 could confer a resistance to anoikis and induce a full transformation of the FE25 cells with a relatively low efficiency.

### Overexpression of CNN1 conferred a flattened and attached cell morphology, reductions in cell motility, invasion, AIG, tumorigenesis, and increase of anoikis

**Figure 4:**
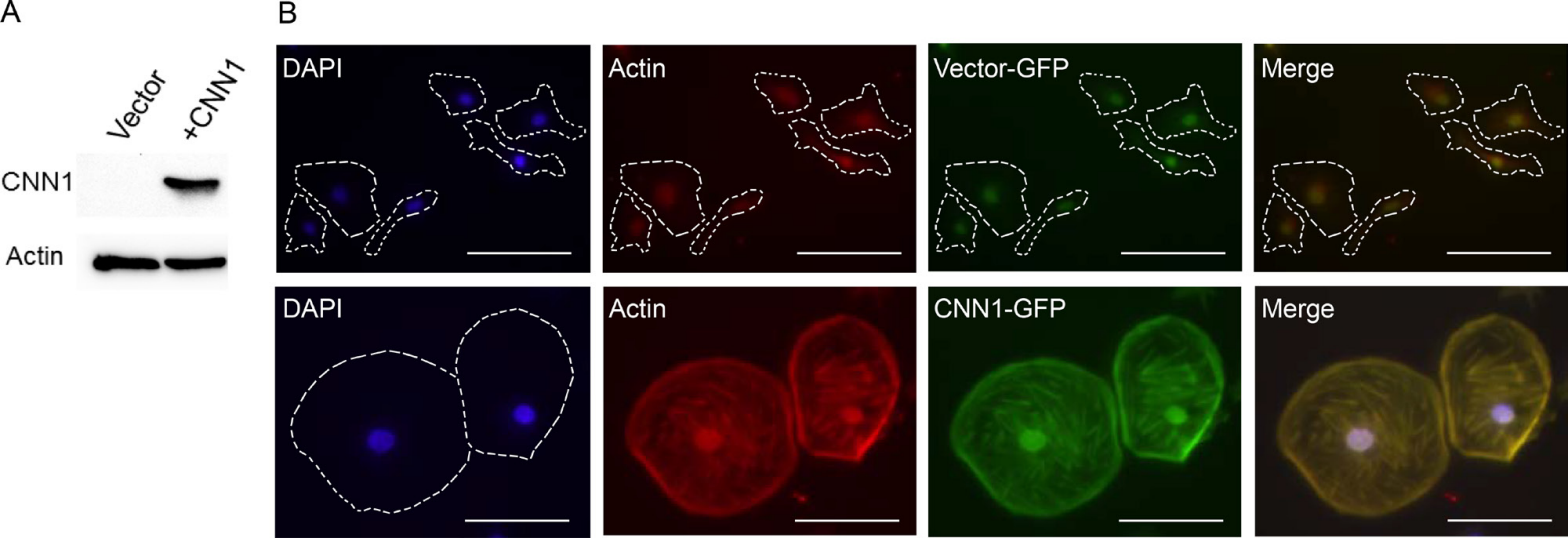
Overexpression of CNN1 in FE-RAS cells confers changes in cell morphology and cytoskeleton distribution (**A**) Western blot of CNN1 in FE-RAS cells transfected with vector and CNN1. (**B**) Immunofluorescence staining of FE-RAS cells transfected with GFP-vector control (upper panel) and GFP-CNN1 (bottom panel). Blue: DAPI. Red: Actin. Green: transfected GFP or GFP-CNN1. The Actin and CNN1 were colocalized in the overlapping field (yellow color). Scale bars: 50 μm. The cell margin was marked with dotted lines.

**Figure 5:**
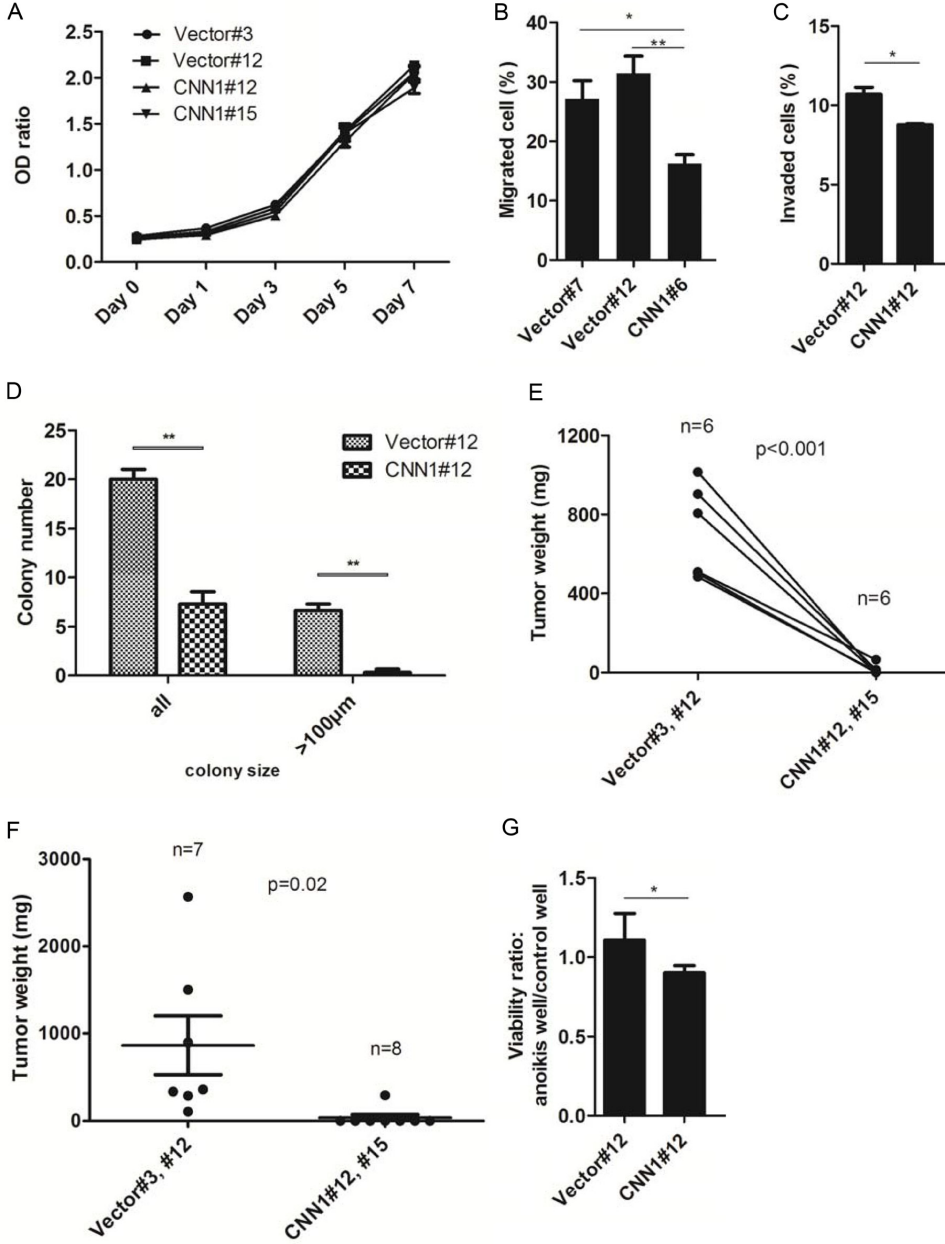
Overexpression of CNN1 in FE-RAS cells results in a suppression of transformation phenotypes (**A**) Cell proliferation of represented clones of FE-RAS cells transfected with CNN1 or vector. (**B**) Transwell cell migration assay of different transfected clones. (**C**) Matrigel invasion assay of represented clones. (**D**) Anchorage-independent growth (AIG) assay. The colonies with size > 50 μm (all) and > 100 μm were counted, respectively. (**E**) Subcutaneous and (**F**) intraperitoneal injection of FE-RAS cells with control vector or stable expressed CNN1 clones. The mice were sacrificed between 6–8 weeks. (**G**) Anoikis assay: cell viability under low attachment culture was tested in FE-RAS cells transfected with control vector or CNN1. *P-value < 0.05, **P-value < 0.01. Showed are results from different reprehensive clones, including clone #3, #7 and #12 of vector control, and clone #6, #12 and #15 of CNN1 transfection. All the multiple clones gave the similar results.

To study the tumor suppressor function of *CNN1*, we cloned the gene into the pEGFP-C1 vector and transfected to the nonexpressing FE-RAS cells (Figure 4A). Compared with the vector-transfected control, the CNN1-overexpressing FE-RAS cells showed dramatic change from a polygonal, raised and slender morphology to a flattened, round and large morphology with prominent actin-stress fibers colocalizing with CNN1 (Figure 4B). Overexpression of CNN1 in the FE-RAS cells did not influence cell proliferation (Figure 5A), but it significantly reduced cell motility (Figure 5B), invasion (Figure 5C), AIG (Figure 5D), and xenograft tumorigenesis (Figure 5E and 5F). In the AIG assay, both the colony number and size were reduced (Figure 5D), and the tumorigenesis in both the subcutaneous and intraperitoneal injection modes in NSG mice was almost completely suppressed (Figure 5E and 5F). The CNN1-overexpressing cells also demonstrated lessened viability in a low-attachment culture, suggesting vulnerability to cell anoikis (Figure 5G).

Compared with cells grown in an attached culture, those grown in a nonattached culture exhibited a lower distribution in the G1 phase (79.8% at 0h vs. 57.5 % at 12 h) and a higher distribution in the G2 phase (7.29% at 0 h vs. 20.7% at 12 h), but overexpression of CNN1 did not alter the cell cycle distribution in either culture form (Supplementary Figure 1).

### The tumor suppressor function of CNN1 is cell type specific

Overexpression of CNN1 in the non-HGSC ovarian cancer cell SKOV3 also showed similar results of suppression of migration, invasion, AIG, and subcutaneous xenograft tumorigenesis. However, unlike the case in the transformed fimbrial cells, neither anoikis resistance nor i.p. tumorigenesis was altered in this clear-cell carcinoma like cell line [29] with CNN1 overexpression (Supplementary Figure 2). These results indicate the tumor suppressor function of CNN1 is cell type specific.

### Re-expression of CNN1 in FE-RAS cells is associated with upregulation of kinase/phosphatase and cytoskeleton genes, and downregulation of cytokine/chemokine genes

To unveil the global gene expressional changes of CNN1 re-expression, we compared the transcriptome of CNN1-transfected FE-RAS cells with that of the vector control cells by using a cDNA microarray. A total of 242 genes were upregulated and 121 downregulated. Among the overrepresenting upregulated genes are those associated with kinase and phosphatase activities and those involved in complex binding of extracellular matrix, cytoskeleton, growth factor, nucleotide and ions (Table 1). The downregulated genes showed an overrepresentation of cytokine and chemokine functions (Table 2). The results suggest that CNN1 overexpression is associated with increases in cell adhesion, scaffolding and signaling, and reductions in cell migration and invasion.

**Table 1:** Summary of increased gene expression in CNN1-overexpressing FE-RAS cells compared with vector-only control

GO number	Term	Number of genes	*P*-value^1^
0004721	Phosphopretein phosphatase activity	9	1.50E-04
0004722	Protein serine/threonine phosphatase activity	5	1.80E-04
0050840	Extracellular matrix binding	4	2.60E-04
0016791	Phosphatase activity	10	7.30E-04
0003707	Steroid hormone receptor activity	4	0.0026
0005520	Insulin-like growth factor binding	3	0.0031
0004879	Ligand-dependent nuclear receptor activity	4	0.0048
0005178	Integrin binding	4	0.0051
0003779	Actin binding	10	0.0053
0004725	Protein tyrosine phosphatase activity	5	0.0077
0019838	Growth factor binding	5	0.008
0032403	Prorein complex binding	7	0.0086
0030145	Manganese ion binding	6	0.0098
0004672	Protein kinase activity	14	0.012
0004674	Protein serine/threonine kinase activity	11	0.013
0008092	Cytoskeletal protein binding	12	0.016
0000166	Nuleotide binding	36	0.029

^1^Modified Fisher exact *P*-value identified by DAVID.

**Table 2:** Summary of decreased gene expression in CNN1-overexpressing FE-RAS cells compared with vector-only control

GO number	Term	Number of genes	*P*-value^1^
0046870	Cadmium ion binding	2	8.00E-04
0008009	Chemokine activity	3	0.001
0042379	Chemokine receptor binding	3	0.0012
0005125	Cytokine activity	5	0.0015
0030955	Potassium ion binding	4	0.0022

^1^Modified Fisher exact *P*-value identified by DAVID.

### Expression of CNN1 confers an increase in cell adhesion to laminin and collagen with integrin α2 β1 partly mediating the cell-laminin adhesion

**Figure 6:**
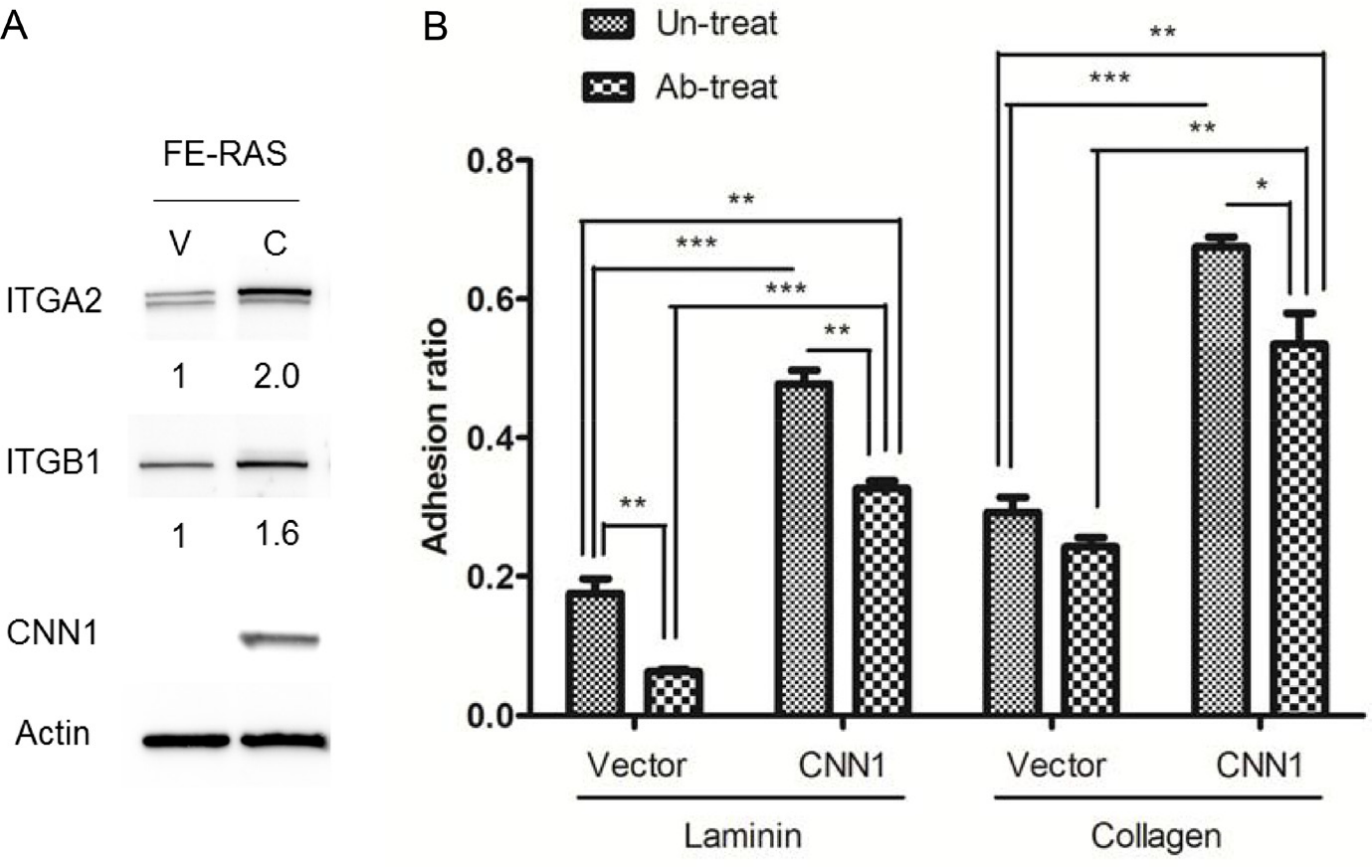
Expressions of integrin α2 (ITGA2) and β1 (ITGB1) correlate with CNN1 expression in cells and confer cell adhesion to different matrixes (**A**) ITGA2, ITGB1 and CNN1 expression level in FE-RAS cells transfected with control vector (clone #12, V) or CNN1 (clone #12, C). The number under each panel indicated the relative quantitation of the band density. (**B**) Cell adhesion assay of different transfectants: 1 × 10^4^ cells were seeded on the laminin- (left panel) or collagen- (right panel) coated well for 30 minutes with or without pre-treatment with integrin α2β1 antibody before seeding, and analyzed for cell adhesion. *p < 0.05, **p < 0.01, ***p < 0.001.

The cDNA microarray results revealed that integrin α2 (ITGA2) and β1 (ITGB1) were apparently upregulated in the CNN1-overexpressing FE-RAS cells. Integrin α2β1 heterodimer is responsible for the cell–extracellular matrix (ECM) interaction and signal transduction in carcinogenesis. As a confirmation, we observed both the α2 and β1 subunit proteins of integrin were upregulated in the CNN1-overexpressing FE-RAS cells (Figure 6A). Compared with the vector control cells, these cells showed a tighter adhesion to the laminin and collagen matrices. Pretreatment with integrin α2β1–blocking antibody significantly inhibited cell adhesion to the collagen matrix in both the vector control and the CNN1-overexpressing FE-RAS cells, whereas the same pretreatment inhibited the adhesion to laminin only in the CNN1-overexpessing cell (Figure 6B). The result indicates that expression of CNN1 in FE-RAS cells is associated with an increase in integrin α2β1 and cell adhesions to the two major components of the basal membrane, and integrin α2β1 is partly responsible for the CNN1-mediated cell-laminin adhesion.

## DISCUSSION

Cancer cells in the *in situ* lesion of STIC are frequently observed to dislodge into the tubal lumen devoid of cell degeneration [8]. The present study reveals that downregulation of the actin-binding protein CNN1 plays an essential role in this early step of metastasis. We determined that overexpression of CNN1 in the transformed FTE cells resulted in recovery to more flattened and attached cell phenotype with an increase in adherence to the basal membrane matrix proteins. In addition, the cells became more vulnerable to anoikis, unable to form a large colony in soft agar, and grew none or only tiny tumor in immune-compromised mice. On contrary, downregulation of CNN1 expression in immortalized FTE cells enhanced anoikis resistance and increased anchorage-independent colony formation, both of which may be related to the survival of exfoliated STIC cells, and became tumorigenic the peritoneum and subcutaneous of NOG mice. Previous studies have revealed an antimetastatic role of CNN1 in both the tumor aspect and the recipient aspects. In CNN1-null transgenic mice receiving intravenous or intraperitoneal injection of B16 melanoma cells, a more extensive metastasis occurred in the lung and in the peritoneal cavity [16, 19]. CNN1 also exerts a tumor suppressor function in mesenchyme tumors such as leiomyosarcoma [21] and fibrosarcoma [24]. In the intraperitoneal spreading model of ovarian cancer, CNN1 acts as a suppressor in both ovarian cancer cells and peritoneal mesothelial cells [19]. This study shows for the first time that CNN1 also plays a critical tumor suppressor role in the non-mesenchymal carcinoma, involving the early event of metastasis of HGSC from the fallopian tube epithelium by maintaining the epithelial architecture and survival within.

CNN1 is reportedly mainly expressed in the smooth muscle cells. We determined that CNN1 was also expressed in the epithelium of the fallopian tube fimbriae. Both the mRNA and protein were readily detected in the primarily cultured and immortalized cells, as well as in the tissue and scrapings of the fallopian tube fimbrial epithelium. In agreement with a tumor suppressor role [21, 24, 25], its mRNA was downregulated by approximately 35-fold in the transformed FTE cells as compared with the nontransformed parental cells. CNN1 was also poorly expressed in HGSC tissues and in the known ovarian HGSC cell lines such as Kuramochi and OVSAHO. Other than the fallopian tube epithelium, CNN1 has never been found in epithelium. We argue that similar to the case of endometrial carcinoma, HGSC may arise from the progenitor cells in the stroma of the fallopian tube mucosa. In the transgenic mouse model with *Dicer* and *Pten* conditionally disabled with *Amhr2-Cre*, high-grade serous carcinomas arose specifically from the fallopian tube stroma as the earliest lesion, suggesting a mesenchymal origin of HGSC [26]. Another genetically engineered ovarian cancer mouse model based on fallopian tube transformation driven by Ovgp promoter also revealed expression of the TAg transgene in the fallopian tube stroma [27].

Interestingly, although CNN1 overexpression caused a suppression of anoikis survival and ip tumorigenesis in transformed FTE cells, the same re-expression in clear cell carcinoma-like SKOV3 cells [29] had no changes of either phenotype. Thus, CNN1 down-regulation may be more important in the development of HGSC than cl cell carcinoma of the ovarian which is known to transform from benign, borderline malignancy to invasive carcinoma.

By binding with actin and related proteins, CNN1 regulates the actin cytoskeleton, affecting the cellular activities of adhesion, migration, and differentiation [19, 24, 28]. In this study, we found that the same role applied to the phenotypic change in the transformation of FTE cells. Motility and matrigel invasion activity decreased and the cell–ECM adhesion increased after its overexpression in the RAS-transformed FTE cells. All of these pointed to a cytoskeleton-stabilizing and transformation-inhibiting function of CNN1.

Downregulation of CNN1 in the immortalized but nontransformed FTE cells resulted in an enhancement of resistance to anoikis and acquired transformation phenotypes of AIG and xenograft tumorigenesis. Sizes of the colonies in soft agar were relatively small, rarely larger than 30 μm, after a 3-week culture. Growth of the xenograft tumors was also slow, taking 5.5 months to reach a detectable size. By comparison, the RAS-transformed FE-RAS cells grew colonies larger than 50 μm, and formed tumors by 1.5 months. A possible explanation is that the turning down of CNN1 may cut down the cytoskeleton-ECM dependency, resulting in a self-autonomous growth of the FE25 cells. With a lack of additional oncogenic driver mutations, such as those involved in PI3K/RAS and NOTCH3 signaling [22], both AIG and *in vivo* tumorigenesis were modest.

To investigate the mechanisms of CNN1 expressional change in carcinogenesis, we examined the global mRNA changes of CNN1 overexpression in the FE-RAS cells. The changes were remarkably monotonous. Only two major groups of genes were upregulated and one group downregulated. Many binding proteins were turned on, suggesting a drastic change in cell scaffolds, including those involved in cytoskeletal protein binding, actin binding, integrin binding, protein complex binding, and extracellular matrix binding. The other group of upregulated genes is associated with signal transduction, including genes involved in nucleotide binding, different kinase activities, different phosphatase activities, different growth factor binding activities, and different nuclear receptor activities (See Table 1 for the different categories of the activities and Supplementary Data for the detail gene list). This list of CNN1-upregulated genes is compatible with the role of CNN1 in stabilizing actin, constituting the static skeletons of cell architecture, and providing a medium for cell signal transduction [10, 15, 29]. Among the limited downregulated genes are chemokines and cytokines (Table 2 and Supplementary Data 1). This suggests that when CNN1 is expressed and the cytoskeleton is stabilized, cell chemotaxis and cell proliferation are suppressed.

We found that expression of the integrin α2β1 subunits, especially integrin β1, was positively correlated with that of CNN1 in the development of fallopian tube HGSC. Both subunit proteins were increased in the CNN1-overexpressing FE-RAS cells. Integrin α2β1 functions as a metastasis suppressor in breast and prostate cancers [30]. Silencing of integrin α2β1 promoted anoikis in breast cancer cells [31]. We also determined that overexpression of CNN1 conferred an increase in cell adhesions to collagen and laminin, two major components of the basal membrane, and that pretreatment with blocking antibody of integrin α2β1 substantially reduced adhesion activities. Specifically, adhesion to laminin was reduced in the CNN1-overexpressing cells but not in the vehicle control, suggesting that integrin α2β1 may partly confer the CNN1-induced cell adhesion.

In summary, this study reveals a tumor suppressor function of CNN1 in the development of ovarian HGSC from fallopian tube fimbriae. Because of its binding to actin and modulation of multiple cellular functions, CNN1 may be the key molecule to be turned down before STIC cells can detach from the fallopian tube epithelium, survive anoikis, and metastasize to the ovarian and peritoneal surface. The study thus identifies a new target for the prevention of ovarian HGSC at its origin.

## MATERIALS AND METHODS

### Clinical specimens and cell culture

Clinical specimens of HGSC and normal tissues were collected from the gynecological oncology service of Tzu Chi General Hospital, Hualien, Taiwan, under the approval of the Institutional Review Board of this hospital. Informed consent was given by each subject. The clinical specimens included 9 HGSC specimens (6 derived from the ovary and 3 from the fallopian tube as the primary site), 6 normal ovarian tissues, 5 normal fallopian tube tissues, and 9 normal fimbriae tissues. Normal fimbrial epithelium was carefully removed using a cytobrush and collected in phosphate buffered saline (PBS). Ovarian cancer cells, OVSAHO, SKOV3, and A1847 were purchased from ATCC and cultured in RPMI1640 and Dulbecco’s modified Eagle’s medium, respectively, supplemented with 10% fetal bovine serum (FBS) and 1% penicillin and streptomycin (PS). The matrigel invasion subclones SKOV3-I6 (six selections for matrigel invasiveness) and A1847-I4 (four selections) were a generous gift from Prof. Lu-Hai Wang of the National Health Research Institutes of Taiwan [32]. Kuramochi cells were purchased from the Japanese Collection of Research Bioresources (JCRB0098) and cultured in RPMI1640 supplemented with 10% FBS and 1% PS.

### Establishment of a transformed human fimbrial epithelial cell line

Primary fimbrial epithelial cells (FTEC) were cultured following the method of Paik DY et al [33]. The methods and characteristics of the HPV16 E6/E7 and hTERT-immortalized FTE cells (FE25 cells) have been described earlier by us [23]. Briefly, human fimbrial epithelium was primarily cultured and transduced with HPV16 E6/E7 lentiviral construct to give rise to FTE cells. The senescence-prone FTE cells were transduced with pLenti-hTERT (Applied Biological Materials, Richmond, BC, Canada) at passage 13 and gave rise to the FE25 cells. The FE25 cells at passage 20 were transduced with pLenti-RasV12 (Applied Biological Materials) to give rise to FE-RAS cells.

### Immunostaining

The xenograft tumors of FE-RAS and clinical specimens of HGSC and normal fimbriae were formalin-fixed and paraffin-embedded, and cut into 4-μm sections. For immunohistochemistry staining, the sections were rehydrated and blocked, and antigens were retrieved through microwave and hydrogen peroxide treatment, after which they were incubated with mouse monoclonal anti-PAX8 antibody (clone PAX8R1, 1:150; Abcam, Cambridge, MA, USA), cytokeratin (clone AE1/AE3, 1:150; Dako, Glostrup, Denmark) or rabbit anti-CNN1 (EP798Y, 1:100; Abcam) for 2 h at room temperature. After washing, the sections were incubated with anti-mouse and anti-rabbit HRP-secondary antibody (Abcam) for 1 h at room temperature and further stained with DAB chromogen (Invitrogen, Carlsbad, CA, USA) and hematoxylin. For immunocytochemistry, FE-RAS cells transfected with GFP vector or GFP-CNN1 were fixed with 4% paraformaldehyde for 20 min, permeabilized with 0.1% Triton X-100 in PBS for 15 min, blocked with 10% FBS for 1 h, incubated with mouse anti-Actin (MM2/193, 1:150; Santa Cruz Biotechnology, Dallas, TX, USA) for 2 h, washed three times, and incubated with Rhodamine Red-X goat anti-mouse IgG (H+L) (Invitrogen) for 2 h and DAPI for 10 min. All the procedures were away from light and at room temperature.

### Quantitative RT-PCR

Total RNA from cell pellets and clinical specimens was extracted using TRIzol reagent (Invitrogen), according to the manufacturer’s instructions. The cDNA was subsequently reverse-transcribed using the RevertAid H Minus First Strand cDNA Synthesis Kit (Thermo Fisher Scientific, Waltham, MA, USA) and further analyzed through quantitative RT-PCR using FastStart Universal SYBR Green Master (Roche, Mannheim, Germany) with the StepOnePlus Real-Time PCR System (Applied Biosystems, Singapore). The primer sequences for CNN1, ITGA2, ITGB1, and ACTB are provided in Supplementary Table 1. The housekeeping gene ACTB was used for endogenous control. The relative expression level was compared with FE25 cells.

### Overexpression of CNN1

The full-length cDNA of CNN1 (NM_001299) was subcloned into pEGFP-C1 vector and further transfected into FE-RAS cells and SKOV3 cells by using Lipofectamine 2000 reagent (Invitrogen). At least five stable clones were picked for the control vector and CNN1-overexpressing cells with neomycin selection. All these clones in either vector or CNN1-transfection group showed the same level of CNN1 expression. The cell morphology was observed under an inverted fluorescent microscope (Zeiss, Oberhocken, Germany). The CNN1 expression pattern was shown by GFP expression and the stress-fiber/cytoskeleton structure was shown by immunocytochemistry staining with actin.

### Knock down of CNN1

The negative control shRNA (shNC) and CNN1 shRNA (shCNN1) (GeneDireX, Miao-Li, Taiwan) were transfected into the FE25 cells by using Lipofectamine RNAiMAX (Invitrogen). The detailed target sequences are shown in Supplementary Table 2. The transfection efficiency was confirmed by inspecting the RFP expression. To obtain high-purity cell clones, each RFP-positive colony was picked using a pipet tip and subsequently cultured under neomycin selection. Three shNC (#1, #2, #3) and four shCNN1 (#3, #4, #5, #10) clones including four different target sites of CNN1 were successfully selected with CNN1 expressional level confirmed.

### Proliferation assay

Cell proliferation assay was performed using 2,3-Bis-(2-Methoxy-4-Nitro-5-Sulfophenyl)-2H-Tetrazolium-5-Carboxanilide (XTT) reagent (Biological Industries, Kibbutz Beit-Haemek, Israel). Briefly, 1 × 10^3^ cells were seeded in a 96-well plate, and the medium was replaced every day for one week experiments. The OD ratio was calculated from the specific absorbance at 450 nm adjusted with a nonspecific absorbance at 650 nm in an ELISA reader (BioTek, Winooski, VT, USA). The blank well contained medium only. Each experiment was triplicated.

### Transwell migration assay

For transwell migration assay, 1 × 10^3^ cells were seeded in the upper channel of a 24-well transwell with an 8-μm pore-sized insert (Falcon, Franklin Lakes, NJ, USA) in serum-free medium. The bottom chamber was loaded with regular medium containing 10% FBS. After 24 h, the cells that migrated to the other side of the transwell were stained and counted. For CNN1 knockdown FE25 cells, 5 × 10^4^ cells were seeded, and the migrated cells were stained using QCM Fluorimetric Cell Migration Assay (Millipore, Temecula, CA, USA) according to the manufacturer’s instructions. Each experiment was triplicated.

### Matrigel invasion assay

For matrigel invasion assay, 5 × 10^4^ cells were seeded on a 24-well plate insert containing extracellular matrix, and the cells that invaded to the bottom of the insert were stained and detected after 24 h by using the QCM ECMatrix Cell Invasion Assay (Millipore) according to the manufacturer’s instructions. The control well contained 5 × 10^4^ cells without matrigel insert. The invasion activity was calculated from the ratio of migrated cells in wells with matrigel insert vs those without. Each experiment was triplicated.

### AIG assay

Sterile 1% (for the bottom layer) and 0.7% (for the top layer) UltraPure LMP low-melting-point agarose (Invitrogen) were mixed with the same volume of medium with 20% FBS. The top layer contained 1 × 10^3^ cells and was incubated for 15–21 days. The cells were fed with 500 μL medium containing 10% FBS every 3 days. Colonies with size > 50 and > 100 μm were each counted in the CNN1-overexpression experiments, and those with size > 20 μm were counted in the CNN1-knockdown experiments. Each experiment was performed in triplicate and each well was randomly selected for five views for counting colony number.

### Xenograft tumorigenesis model

Cells were subcutaneously and intraperitoneally injected into NOD.Cg-Prkdc(scid)Il2rg(tm1Wjll)/SzJ, abbreviated NOD/scid/IL-2Rgamma null (NSG) mice [34]. For the subcutaneous injections, the experimental CNN1-overexpressing (or knockdown) cells and control cells were each injected into the two flanks of the same mouse. For the intraperitoneal injections, cells were individually inoculated. The cell number of each injection was 1 × 10^6^ cells in 100 μL PBS. Mice for the FE-RAS (CNN1-overexpressing) experiments were sacrificed between 6 and 8 weeks, and those for the FE25 (CNN1-knockdown) experiments were sacrificed at 5.5 months. All the tumors were collected and weighed.

### Anoikis assay

The anoikis activity of the cells was investigated using a CytoSelect 96-well anoikis assay kit (Cell Biolab, San Diego, USA). Briefly, 1 × 10^4^ cells were seeded onto the nonadherent poly-HEMA-coating well under regular culture conditions, and cell viability was detected 24 h later using XTT reagent (Biological Industries). The control well contained 1 × 10^4^ cells without poly-HEMA coating. The viability ratio of cells grown in the two different wells was calculated using OD_anoikis well_/OD_control well_. Each experiment was triplicated.

### Western blotting

Cell lysates were collected and proteins were purified using radioimmunoprecipitation assay buffer (Thermo Fisher Scientific) containing protease and phosphatase inhibitors (Roche). Extracts (30 μg) were loaded into sodium dodecyl sulfate–polyacrylamide gel electrophoresis (SDS-PAGE) and transferred onto a polyvinylidene difluoride (PVDF) membrane by using the iBlot Dry Blotting System (Life Technologies, Hertzliya Pituach, Israel). After blocking with skim milk for 1 h, incubation with primary antibody overnight, and washing with PBST (PBS with 0.05% Triton-X100) three times, we incubated the membrane with a secondary HRP-linked antibody for another hour. The signal was visualized using enhanced chemiluminescence reagent (Millipore) under the Gel Documentation System (ANT Technology Co., Taipei, Taiwan), and the density was quantified using ImageJ software (National Institutes of Health, Bethesda, Maryland, USA). The primary antibodies included rabbit anti-p53 monoclonal antibody (#2527, 1:1000; Cell Signaling Technology, Danvers, MA, USA), mouse monoclonal anti-RAS (1:1000; Abcam), mouse monoclonal anti-PAX8 (clone PAX8R1, 1:1000; Abcam), mouse monoclonal anti-HPV16+HPV18 E6 (C1P5, 1:1000; Abcam), mouse monoclonal anti-HPV16 E7 (289–17013, 1:1000; Abcam), rabbit anti-CNN1 (EP798Y, 1:2500; Abcam), mouse anti-Actin (MM2/193, 1:10000; Santa Cruz Biotechnology), rabbit anti-integrin alpha 2/CD49b (1:1000; Bioss, Woburn, MA, USA), and rabbit anti-integrin beta 1 (1:1000; Bioss). The secondary antibodies included rabbit anti-mouse IgG H&L HRP (1:20000; Abcam) and goat anti-rabbit IgG H&L HRP (1:10000; Abcam).

### Microarray analysis

Gene expression profiling was conducted with the Human OneArray (Phalanx Biotech Group, Hsinchu, Taiwan) containing 28,264 human genome probes. We compared the CNN1-overexpressing FE-RAS cells with the vector-transfected control FE-RAS cells. The 242 upregulated and 121 downregulated genes (Supplementary Data 2) were subjected to gene ontology analysis using the DAVID online functional annotation tool at https://david.ncifcrf.gov/home.jsp. Statistical significance was included when *P* < 0.05.

### Cell adhesion assay

For cell adhesion assay, a 96-well plate was coated with 10 μg/mL of laminin (Invitrogen) or collagen (Sigma-Aldrich, St. Louis, MO, USA) for 2 h at room temperature and then removed by inverting the plate and washing twice with PBS. After the wells were blocked with regular medium containing 10% FBS for 1 h in an incubator, 1 × 10^4^ cells were seeded and cultured for 30 min. The wells were then inverted to pour out nonadhesive cells and then washed with PBS. The remaining adhesive cells were fed with fresh medium and the viability was determined using XTT. An equal number of cells cultured on a nontreated plate served as the control. The ratio of viable adhesive cells was calculated using OD_adhesive_/OD_control_.

For the functional test of integrin α2β1, we pretreated FE-RAS cells with mouse monoclonal antibody of integrin α2β1 (P1E6, 5μg/mL; Abcam) for 30 min, and followed the experimental procedures above. Each experiment was triplicated.

### Statistical analysis

All statistical analyses were performed using Prism 5.0 (GraphPad Software, San Diego, CA, USA). A two-tailed Student’s *t* test was used to analyze the experimental data. Statistical significance was set at *P* < 0.05. Data are presented as mean ± standard error.

## SUPPLEMENTARY MATERIALS FIGURES AND TABLES



## SUPPLEMENTARY MATERIALS DATA




