# Supplementary material for: Loss of calponin h1 confers anoikis resistance and tumor progression in the development of high-grade serous carcinoma originating from the fallopian tube epithelium
Source: Oncotarget. 2017 May 19;8(37):61133–45. doi: 10.18632/oncotarget.18024 (PMC5617412; doi:10.18632/oncotarget.18024)
Supplement: Supplementary file 1 [file oncotarget-08-61133-s001.pdf]

Loss of calponin h1 confers anoikis resistance and tumor progression in the development of high-grade serous carcinoma originating from the fallopian tube epithelium

SUPPLEMENTARY MATERIALS

Supplementary Table 1: The primers used in this study

Target	Sequence
CNN1 QF	GCGGAAATTCGAGCCGGGGAA
CNN1 QR	TGTGCCCAGCTTGGGGTCGT
CNN1-Full-F	GGATCCATGTCCTCTGCTCACTTC
CNN1-Full-R	GGTACCTAGGCGGAATTGTAGTAG
ACTB QF	AACTCCATCATGAAGTGTGACG
ACTB QR	GATCCACATCTGCTGGAAGG

Supplementary Table 2: The control and CNN1 shRNA used in this study

Target	Sequence
GAPDH	GTATGACAACAGCCTCAAG
NC	TTCTCCGAACGTGTCACGT
CNN1-322	GCCTCAAAGATGGCATCATTC
CNN1-416	GCTGGAGAACATCGGCAACTT
CNN1-555	GCGAAGACGAAAGGAAACAAG
CNN1-1032	GCACACAACTACTACAATTCC

Backbone vector: PU6/RFP/Neo.
The numbers are the position of nucleotide of CNN1 mRNA.

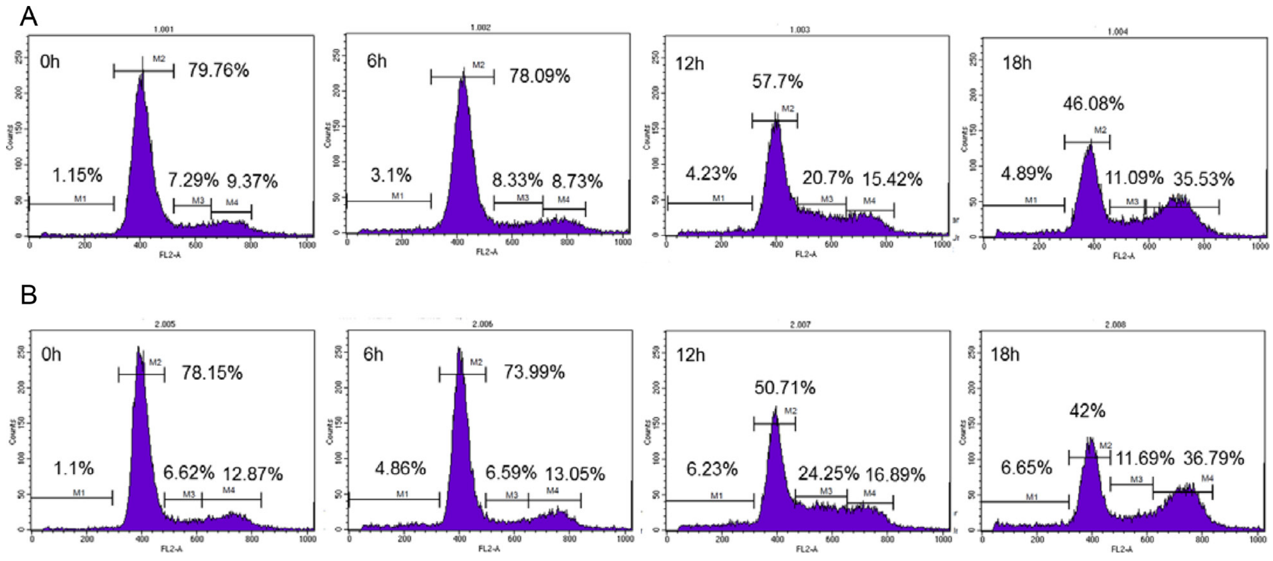

Supplementary Figure 1: Anoikis culture resulted in a reduced distribution at G1 phase and increase distribution at G2 phase of cell cycle in FE-RAS cells regardless the level of CNN1 expression. (**A**, **B**) Cell cycle analysis of FE-RAS transfected with control vector clone #12 (A) or CNN1 clone #12 (B) and grew in poly-HEMA coating plate for 0 h, 6 h, 12 h, and 18 h. The percentage of cells at sub G0, G1, S, G2 phases were showed, respectively.

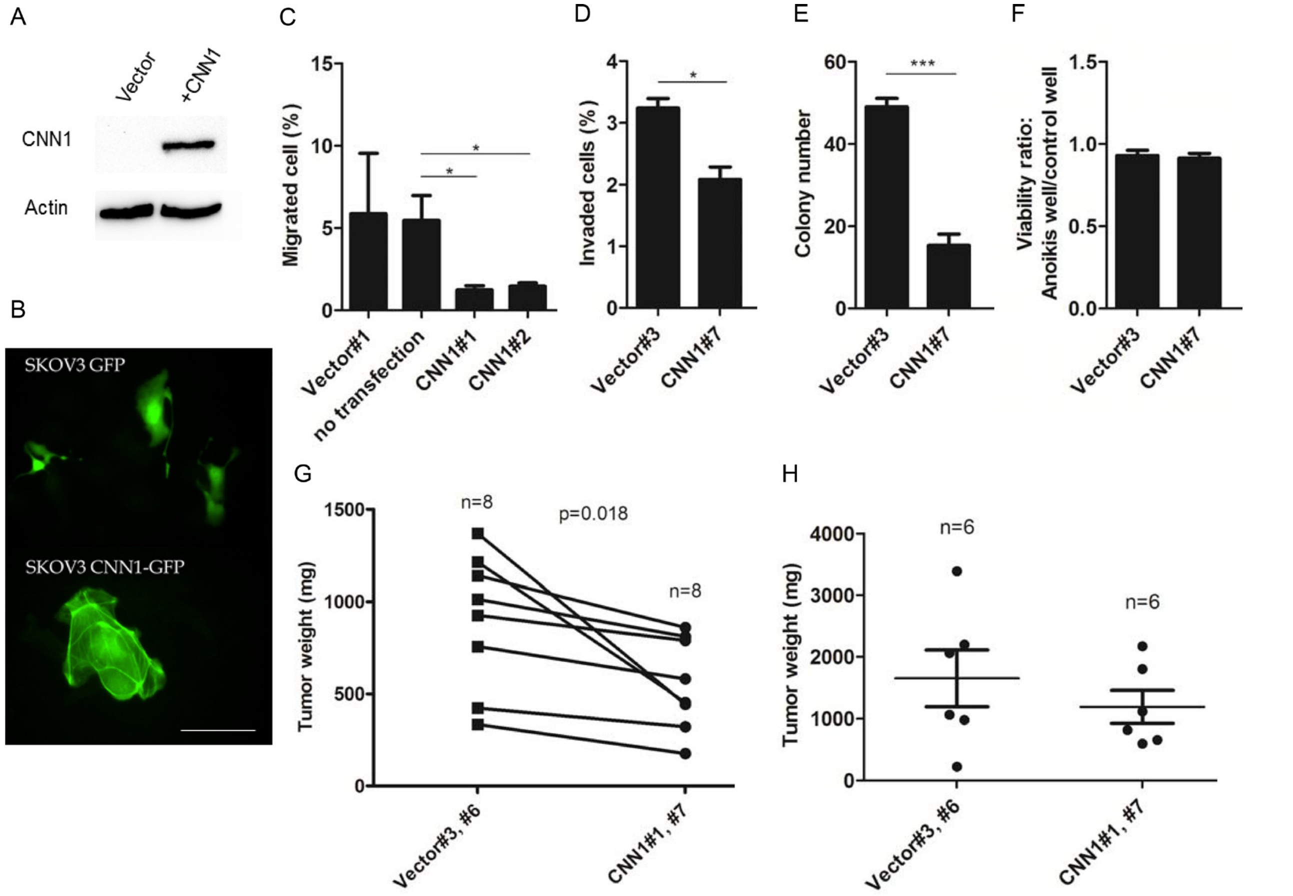

Supplementary Figure 2: Overexpression of CNN1 conferred a change of cellular morphology and suppression of transformation phenotype of SKOV3 cells. CNN1 (+CNN1) and the GFP-vector only (Vector) were transfected into SKOV3 cells. Expression of CNN1 protein was confirmed by Western blot (**A**). Morphology of the GFP vector- and CNN1-transfectants viewed under inverted fluorescent microscope (**B**). Comparisons of transformation phenotypes including cell migration (**C**), invasion (**D**), anchorage-independent growth (AIG) (**E**), survival to anoikis (**F**), as well as xenograft tumorigenesis with s.c. (**G**) or i.p. (**H**) injection were showed. Showed are results from representative clones. Detail materials and methods were the same as shown in the article.

Supplementary Data 1: Microarray data of CNN1-overexpressing vs vector-control FE-RAS cells. See Supplementary_Data_1.

Supplementary Data 2: Gene list after ontology analysis. See Supplementary_Data_2.
